# Scalable Synthesis Nano-Perovskite K(Mn_0.95_Ni_0.05_)F_3_ Cathode by Homogeneous Precipitation Method for Potassium-Ion Batteries

**DOI:** 10.1186/s11671-019-3056-1

**Published:** 2019-07-16

**Authors:** Shuya Wang, Bin Cui, Quanchao Zhuang, Yueli Shi, Hong Zheng

**Affiliations:** 1 Key Laboratory of Synthetic and Natural Functional Molecule of Ministry of Education, Shanxi Key Laboratory of Physico-Inorganic Chemistry, College of Chemistry and Materials Science, Northwest University, Xi’an, 710127 China; 2 Qinghai Engineering and Technology Research Center of Comprehensive Utilization of Salt Lake Resources, Qinghai Institute of Salt Lakes, Chinese Academy of Sciences, Xining, 810008 China; 3 Li-ion Batteries Lab, School of Materials Science and Engineering, China University of Mining and Technology, Xuzhou, 221116 China; 4 Department of Chemistry, Chongqing Normal University, Chongqing, 401331 China

**Keywords:** Concentration-gradient structure, Electrochemistry, Homogeneous precipitation, Nanoparticles, Potassium-ion batteries

## Abstract

Potassium-ion batteries (KIBs) are favored by researchers because of the unique advantages. In this work, KIB cathode material nano-perovskite K(Mn_0.95_Ni_0.05_)F_3_ with concentration gradient was synthesized by EDTA-assisted homogeneous precipitation method for the first time and characterized. The solid solution material was deposited on the multi-walled carbon nanotubes (MWCNTs) to form K(Mn_0.95_Ni_0.05_)F_3_/MWCNT nanocomposites to improve the electron conductivity of the electrode material so as to obtain the excellent electrochemical performance. As expected, the charge and discharge capacities of K(Mn_0.95_Ni_0.05_)F_3_/MWCNTs after the 60th cycle can still reach 106.8 and 98.5 mAh g^−1^ over the voltage range 4.2–1.2 V vs. K/K^+^ at the current density of 35 mA g^−1^, respectively. Electrochemical performance studies showed that solid solution K(Mn_0.95_Ni_0.05_)F_3_ had the potential applications as the cathode material for KIBs. Electrochemical impedance spectroscopy (EIS) was used to study the transport and reaction processes of ions at the solid–liquid interface. The main factors affecting electrochemical performance could be analyzed from the Nyquist plot of the EIS test.

**Electronic supplementary material:**

The online version of this article (10.1186/s11671-019-3056-1) contains supplementary material, which is available to authorized users.

## Introduction

Driven by the growing demand for portable instruments and devices, a wide range of research groups have engaged in comprehensive and in-depth research on lithium-ion batteries (LIBs) [1, 2]. The application of LIBs is limited because of the relative rarity and uneven distribution of lithium resources [3, 4]. Potassium, as the same main group and adjacent element after sodium, can provide a lower reduction potential, allowing it to be operated at higher potentials to increase the energy density. Compared with sodium-ion batteries (NIBs), potassium-ion batteries (KIBs) are less studied and still in the early stage of development, especially the cathode material [5, 6].

The critical point to develop excellent performance KIBs mainly lies in designing the rational microstructure of the cathode materials to realize the ideal insertion/extraction of K-ion. In the current application field for KIBs, vanadium-based and open framework cathode have received extensive attention due to the high-voltage platform and accommodating corresponding volume changes during the charge and discharge cycle, respectively [7–11].

From the perspective of a wide range of costs and resources, manganese-based materials have received extensive attention as electrode materials for various types of batteries, such as lithium-ion batteries [12], sodium-ion batteries [13, 14], and flow batteries [15]. Among them, manganese-based layered oxides are favored by researchers due to their high theoretical capacity [13]. However, as the cathode of KIB, manganese-based layered oxides exhibit limited capacity and a relatively low voltage platform, which limits their application [16]. It has been reported that representative cathode in manganese**-**based material K_0.3_MnO_2_ [17] and K_0.5_MnO_2_ [18] do not achieve a charging voltage higher than 4 V. In order to enrich the research of manganese-based electrode materials for KIBs, other types of manganese-based electrode materials have also received increasing attention.

Based on the resource advantages of manganese and our team’s work in the study of fluoride cathode materials, we choose perovskite manganese-based fluoride as the base cathode material [19–21]. Fluorine-containing electrode materials have high pressure resistance and can alleviate the defects of low voltage platform of electrode materials [22]. The major reason to limit the application of fluorine-containing is the strong ionic bond characteristics of fluoride resulting in a wide band gap and poor electron conductivity [23]. An efficient way to facilitate the charge transfer process of the electrode material is to rationally design composites [24]. The existing methods for preparing fluoride suffer from a number of notable limitations, such as the requirement of an ultrahigh temperature, complex procedures and the usage of corrosive HF and toxic F_2_ [20, 25]. The homogeneous precipitation method has been successfully applied to the preparation of other electrode materials and has achieved excellent electrochemical performance [26]. This method has the advantages of mild synthesis conditions, uniform particle size preparation and controllable morphology. Therefore, if the homogeneous precipitation method is used to synthesize a manganese-based fluoride nanomaterial, it is desirable to simultaneously solve the problem of the harsh preparation conditions and the poor conductivity. On the one hand, the fluoride with uniform particle size can effectively mix with conductive material to form a composite phase, and then improve the overall conductivity of the electrode material [27–30]. On the other hand, the effect of improving the conductivity of the material can be achieved by using more internal electron channels and tunneling effect of nanomaterials with special morphology [31, 32].

In this paper, nano-perovskite cathode material K(Mn_0.95_Ni_0.05_)F_3_ was synthesized by EDTA-assisted homogeneous precipitation method. EDTA acts as a buffer and chelating agent to control the release rate of Mn during precipitation [33, 34]. In addition, EDTA prevents particle coagulation by shielding the metal ions, which is another necessity for the preparation of monodispersed particles [35]. Nano-structure can increase the surface reactivity and shorten electronic and ionic pathways within particles [36–38]. To this end, nano-perovskite K (Mn_0.95_Ni_0.05_)F_3_ was used as the cathode for KIBs. Meanwhile, K(Mn_0.95_Ni_0.05_)F_3_/MWCNT nanocomposite electrode material obtained superior electrochemical performance by nano-level mixing of the active material and the conductive agent. Electrochemical impedance spectroscopy (EIS) was used to study the transport and reaction processes of ions at the solid-liquid interface.

## Materials and Methods

### Raw Material

The raw materials are listed as follows: C_10_H_14_N_2_O_8_Na_2_·2H_2_O (EDTA-2Na, 98%, Aladdin), Mn(CH_3_COO)_2_·4H_2_O (99%, Aladdin), Ni(CH_3_COO)_2_·4H_2_O (99.9%, Aladdin), KF (99%, Aladdin), multi-walled carbon nanotubes (MWCNTs; > 95%, Aladdin), polyvinylidene fluoride (PVDF; Arkema), and *N*-methyl pyrrolidone (NMP; 99%, Macklin).

### Material syntheses

>Nano-perovskite K(Mn_0.95_Ni_0.05_)F_3_ was synthesized using a new synthesis method, namely EDTA-assisted homogeneous precipitation. All reagents used were of analytical grade and were used directly without any purification. The synthetic steps were shown below. Six millimoles of EDTA-2Na and 5.25 mmol of Mn(CH_3_COO)_2_·4H_2_O were dissolved in 75 ml of water and 75 ml of ethanol, stirred and dissolved. Then, 20 mmol of KF was added and dissolved, and the resulting solution was named A. Ni(CH_3_COO)_2_·4H_2_O (6.0 mmol, 6.25 mmol, and 6.5 mmol) was dissolved in 80 mol of water and 80 ml of ethanol and added dropwise to solution A using a dropping funnel under continuous stirring. Under the conditions of reacting for 30 min and standing for 12 h, the production was centrifuged using high-speed centrifuge (Biobase, TD-4 M, Jinan, China) to obtain a solid product Then, the solid product was washed several times with ethanol and distilled water, collected and dried at 60 °C to obtain KMnF_3_, K(Mn_0.975_Ni_0.025_)F_3_ and K(Mn_0.95_Ni_0.05_)F_3_, respectively. The synthesis of KMnF_3_ and K(Mn_0.975_Ni_0.025_)F_3_ was used to compare and verify the formation of K(Mn_0.95_Ni_0.05_)F_3_.

### Fabrication of K(Mn0.95Ni0.05)F3/MWCNT Composite

MWCNTs (0.1 g) were added directly to 25 ml of water and ethanol (volume ratio, 1:1) at room temperature and sonicated for 0.5 h to achieve good dispersion. The dispersed carbon nanotubes were added to the solution A and stirred. The subsequent steps were performed according to the same procedure as the syntheses of K(Mn_0.95_Ni_0.05_)F_3_.

### Material Characterization

The structure and chemical components of the products were characterized by X-ray diffraction (XRD; Bruker D8 ADVANCE with Cu Kα radiation) over an angular range of 10–70° with a step width of 0.02° (40 KV, 40 mA) and X-ray photoelectron spectroscopy (XPS; ESCALAB 250Xi with 150 W Al Ka probe beam). The morphology of the synthesized products was analyzed by field mission transmission electron microscopy (Tecnai G2 F20). The exact element content of the prepared materials was determined by inductively coupled plasma atomic emission spectrometry (ICP-AES; Thermo Scientific iCAP 6500 Duo).

### Electrochemical Characterization

To prepare working electrodes,70 wt% active material (K(Mn_0.95_Ni_0.05_)F_3_, K(Mn_0.95_Ni_0.05_)F_3_/MWCNTs), 20 wt% conductive agent, and 10 wt% PVDF binder in NMP solvent were ball-milled in a planetary ball mill (Nanjing University Instrument Factory, QM-3SP04, Nanjing, China) to achieve thorough mixing and coated on the aluminum foil. The prepared electrode film was dried under vacuum at 120 °C for 12 h. The electrolyte was 0.85 mol L^−1^ KPF_6_ in ethylene carbonate (EC) and diethyl carbonate (DEC) (1:1, *v*/*v*; Mojiesi Energy Technology Co., Ltd., Nanjing, China). The button battery was assembled in a glove box with argon atmosphere (Mikrouna super 1220/750, Shanghai, China). The assembled battery was used to test the charge and discharge progress of the KIBs in the battery analyzers (Neware, Shenzhen, China) over a range of 4.2–1.2 V vs. k/k^+^. EIS was tested on an electrochemical workstation (CHI660D, Chenhua Co., Ltd, Shanghai, China) using a three-electrode system with a frequency range from 10^5^ to 10^−2^ Hz.

## Results and Discussion

### Structural and Morphological Characterization of K(Mn0.95Ni0.05)F3 Nanoparticles

**Fig. 1:**
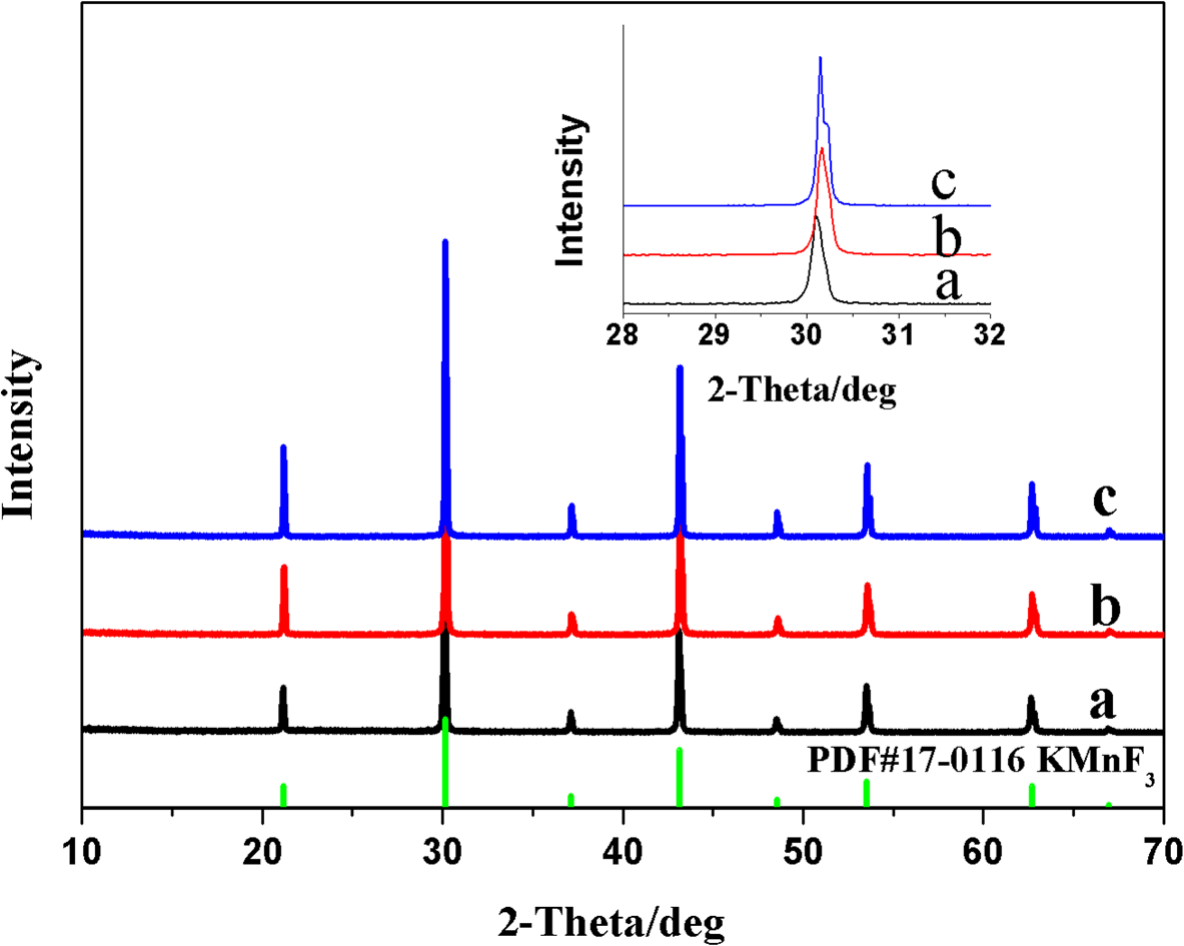
The XRD patterns of KMnF_3_ (a), K(Mn_0.975_Ni_0.025_)F_3_ (b), and K(Mn_0.95_Ni_0.05_)F_3_ (c) corresponding to different nickel acetate additions (a, b, and c represent the corresponding products when the addition of nickel acetate were 6.0 mmol, 6.25 mmol, and 6.5 mmol, respectively)

XRD patterns could be used to confirm the formation of the solid solution K(Mn_0.95_Ni_0.05_)F_3_. Figure 1 showed the XRD pattern of the product at different nickel acetate additions. When the amount of nickel acetate added was 6 mmol, all of the nickel ions participated in the complexation reaction to form EDTA-Ni, and the product was a pure perovskite structure KMnF_3_ (PDF 17-0116). This result confirmed that the manganese ions displaced in EDTA-Mn participate in the precipitation reaction at the beginning of the reaction. When the nickel acetate addition continued to increase to 6.25 mmol and 6.5 mmol, the diffraction peak gradually shifted to a higher angle to form K(Mn_0.975_Ni_0.025_)F_3_ and K(Mn_0.95_Ni_0.05_)F_3_, respectively. This phenomenon was mainly due to the partial substitution of Ni^2+^ with smaller ionic radius for Mn^2+^ with larger ionic radius to form a solid solution structure. ICP-AES was used to further determine the elemental ratio of manganese-cobalt in K(Mn_0.975_Ni_0.025_)F_3_ and K(Mn_0.95_Ni_0.05_)F_3_. The calculation results were close to the theoretical ratio according to the amount added in the synthesis process (Table 1).

**Table 1:** Test results of ICP-AES of synthetic material K(Mn_0.975_Ni_0.025_)F_3_ and K(Mn_0.95_Ni_0.05_)F_3_

Electrode	Concentration of Mn/mmol L^−1^	Concentration of Co/mmol L^−1^	Measured molar ratio of Mn to Co	Theoretical molar ratio of Mn to Co
K(Mn_0.975_Ni_0.025_)F_3_	0.1289	0.0032	40.28:1	39:1
K(Mn_0.95_Ni_0.05_)F_3_	0.1252	0.0067	18.70:1	19:1

**Fig. 2:**
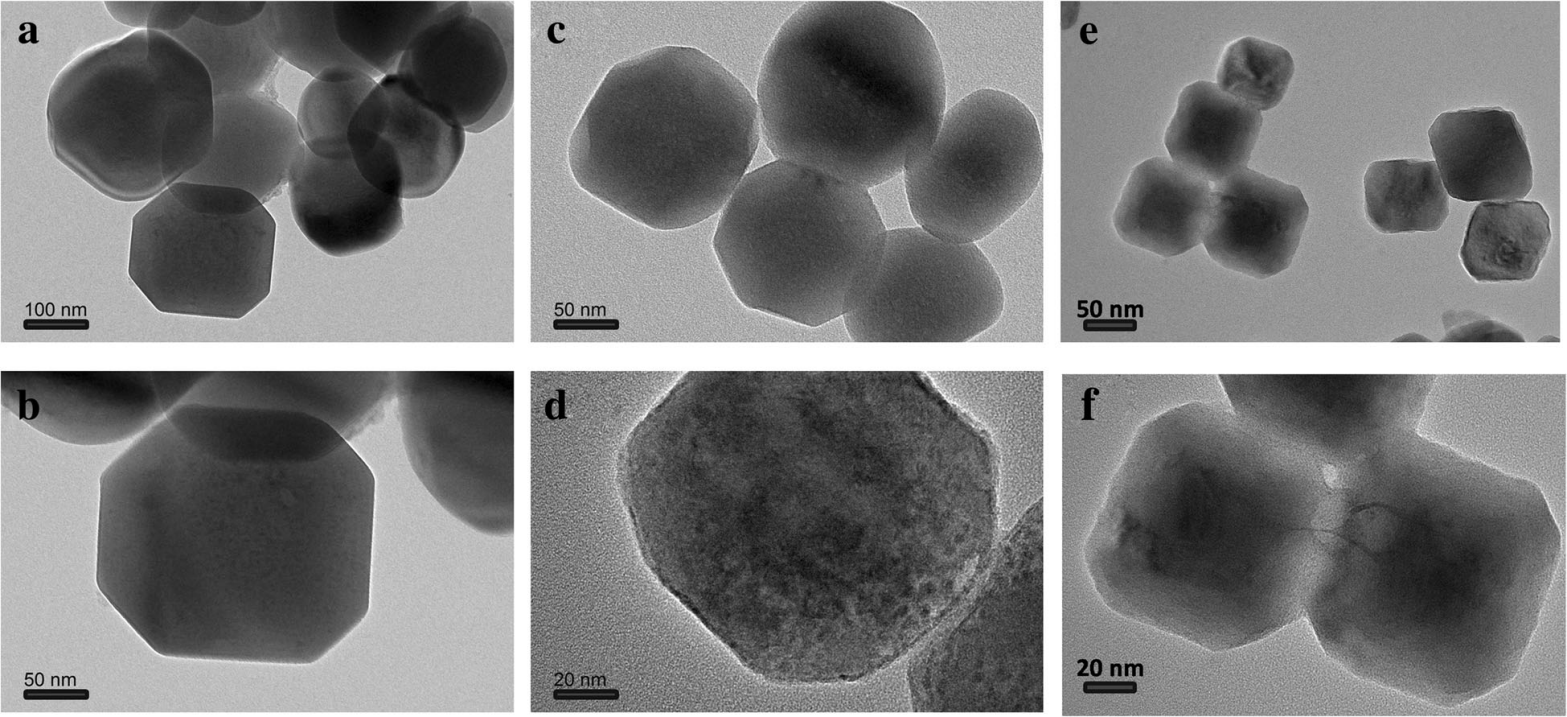
TEM images of KMnF_3_ (**a**, **b**), K(Mn_0.975_Ni_0.025_)F_3_ (**c**, **d**), and K(Mn_0.95_Ni_0.05_)F_3_ (**e**, **f**)

Figure 2 showed the TEM images of KMnF_3_, K(Mn_0.975_Ni_0.025_)F_3_, and K(Mn_0.95_Ni_0.05_)F_3_, which matched the XRD results. Since the release rate of Mn was controlled by using EDTA as a buffer and a chelating agent in the precipitation process, the prepared particles had good particle dispersion and uniform particle size. As shown in Fig. 2a, b, the product KMnF_3_ nanoparticles showed an average size of about 150 nm and an uneven particle size distribution. As shown in Fig. 2c, d, the size average particle size of K(Mn_0.975_Ni_0.025_)F_3_ nanoparticles was about 120 nm, which was significantly less than KMnF_3_ nanoparticles. As depicted in Fig. 2e, f, the uniform dispersed K(Mn_0.95_Ni_0.05_)F_3_ nanoparticles showed an average size of about 100 nm. What was more worth mentioning was that the reduction in particle size did not affect the retention of good dispensability. Nanoparticles with the narrow particle size distribution could improve the contact between the particles and the conductive agent and shorten the electron and ion pathways within the particles, thereby increasing the electrical conductivity. Significant contrast changes of the nanoparticles from the inside to the outside could also be seen from the TEM images.

In view of the smaller particle size and uniform particle size distribution of the solid solution structure K(Mn_0.95_Ni_0.05_)F_3_, it was selected as the research object for subsequent further morphological and performance characterization.

**Fig. 3:**
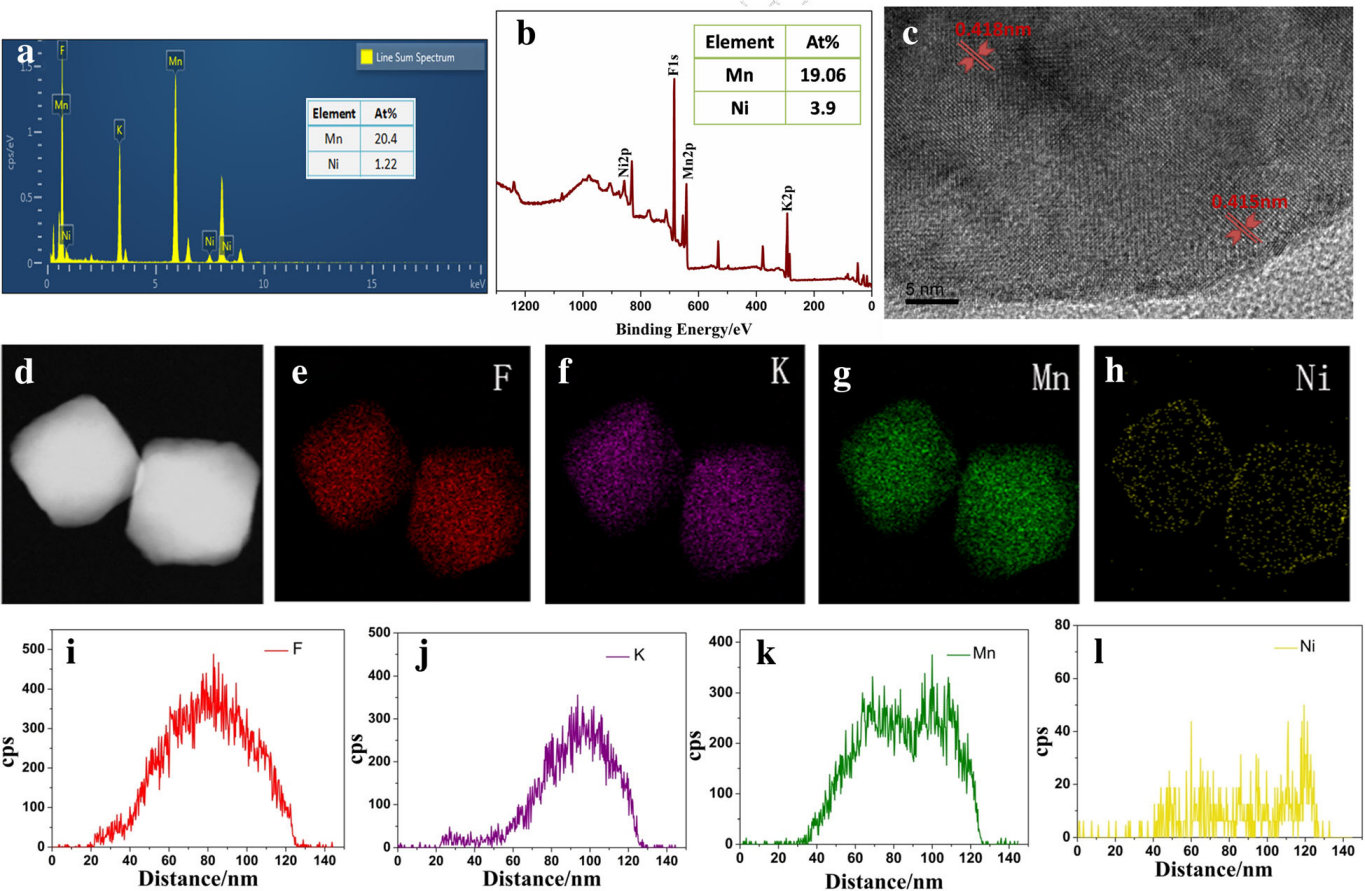
The further supplement and verification of the structure and morphology of K(Mn_0.95_Ni_0.05_)F_3_nanoparticles (**a** corresponds to EDS image; **b** corresponds to XPS image; **c** corresponds to HRTEM images; **d** corresponds to the electron image; **e**, **f**, **g**, and **h** correspond to F, K, Mn, and Ni elements of mapping images, respectively; **i**, **j**, **k**, and **l** correspond to F, K, Mn, and Ni elements of line scanning curves, respectively)

**Scheme 1:**
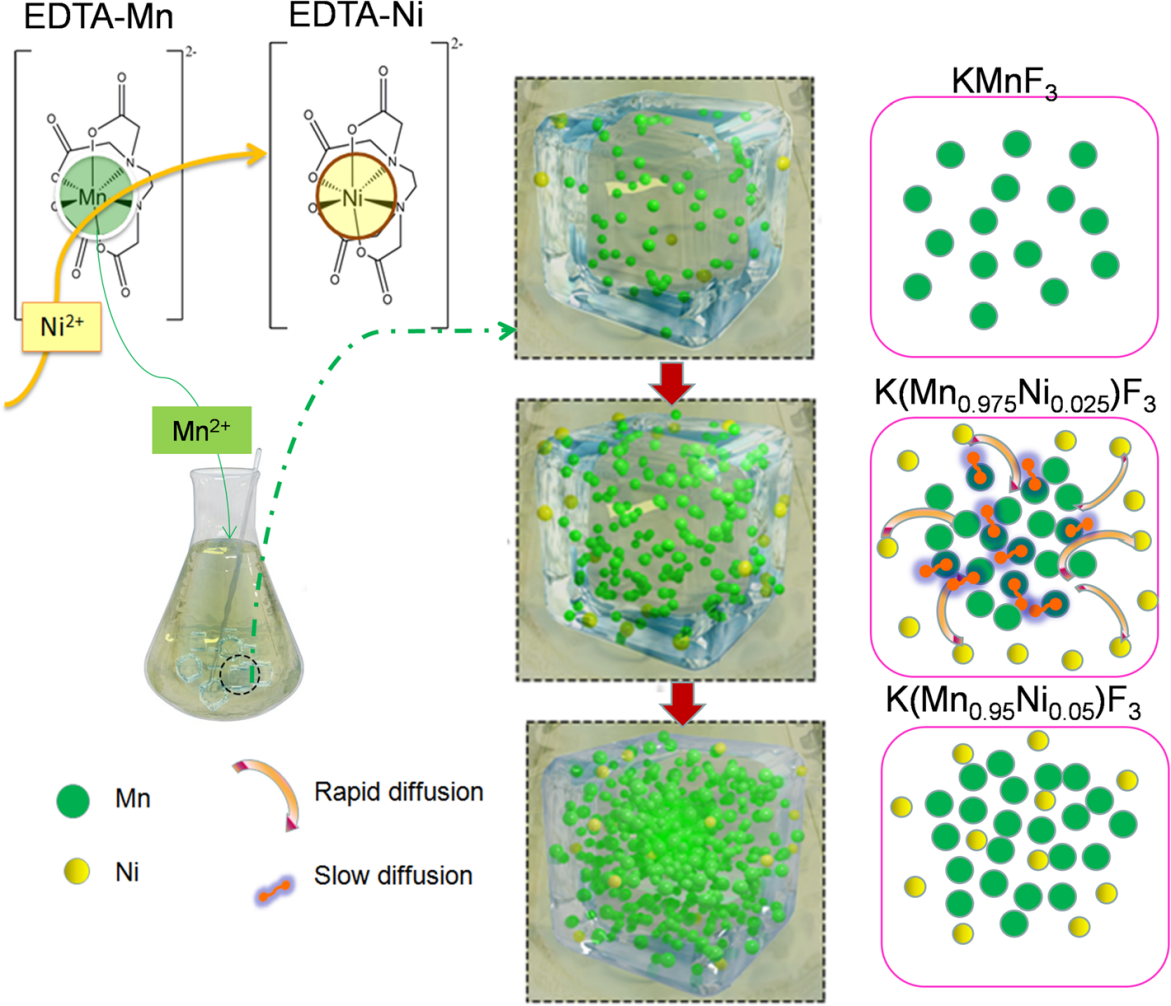
Synthesis process of the concentration-gradient structure of K(Mn_0.95_Ni_0.05_)F_3_

The further supplement and verification of the structure and morphology of K(Mn_0.95_Ni_0.05_)F_3_ was demonstrated in Fig. 3. The energy-dispersive spectrum (EDS) further verified the elemental composition and the element ratio of Mn to Ni of the synthesized nanoparticles K(Mn_0.95_Ni_0.05_)F_3_, as shown in Fig. 3a, b. As can be seen from Fig. 3c, the change in interplanar spacing was also used to aid in demonstrating the structure of the particles. The interplanar spacing of 0.418 nm matched the (100) crystal plane of KMnF_3_ (PDF 17-0116), and the interplanar spacing of 0.415 nm matched the K(Mn,Ni)F_3_ solid solution. In addition, elemental mapping images (d, e, f, g, h) and the line scanning curves (i, j, k, l) in Fig. 3 suggested the corresponding distribution of F, K, Mn, and Ni elements for the K(Mn_0.95_Ni_0.05_)F_3_ solid solution structure. As can be seen from the mapping and line scan results of Mn and Ni elements, the elemental distribution of Ni was relatively uniform, while that of Mn element was more distributed in the center of the particle, decreasing gradually from the center to the surface. The elemental distribution of Mn in nanoparticles showed the significant concentration gradient. The predicted synthesis process of the concentration-gradient structure was given in Scheme 1. At the beginning of the reaction, Mn^2+^ in EDTA-Mn was slowly replaced by Ni^2+^ and first participated in the reaction. With the increasing of the reactions, Ni^2+^ participated in the reaction and coated the surface of the particles. Ni^2+^ in the surface of the reaction prevented the diffusion of Mn^2+^ during the reaction. The difference in diffusion rate of Mn^2+^ and Ni^2+^ leaded to the formation of the concentration-gradient structure. In addition, the surface content of Ni element from the XPS tests (Additional file 1: Figure S1) was relatively higher than that of the EDS test, which was also an auxiliary proof of the concentration-gradient structure.

The special structure of K(Mn,Ni)F_3_ could effectively prevent the dissolution of manganese ions in the electrolyte and improve the cycle stability of the potassium-ion batteries. The concentration-gradient structure could effectively realize the ion migration and electron transfer during charge–discharge, leading to superior electrochemical properties [39]. Another advantage that cannot be ignored was that the concentration-gradient structure can overcome the shortcomings of the structure mismatch in the general core–shell electrode [24].

### Structural and Morphological Characterization of K(Mn0.95Ni0.05)F3/MWCNTs

**Fig. 4:**
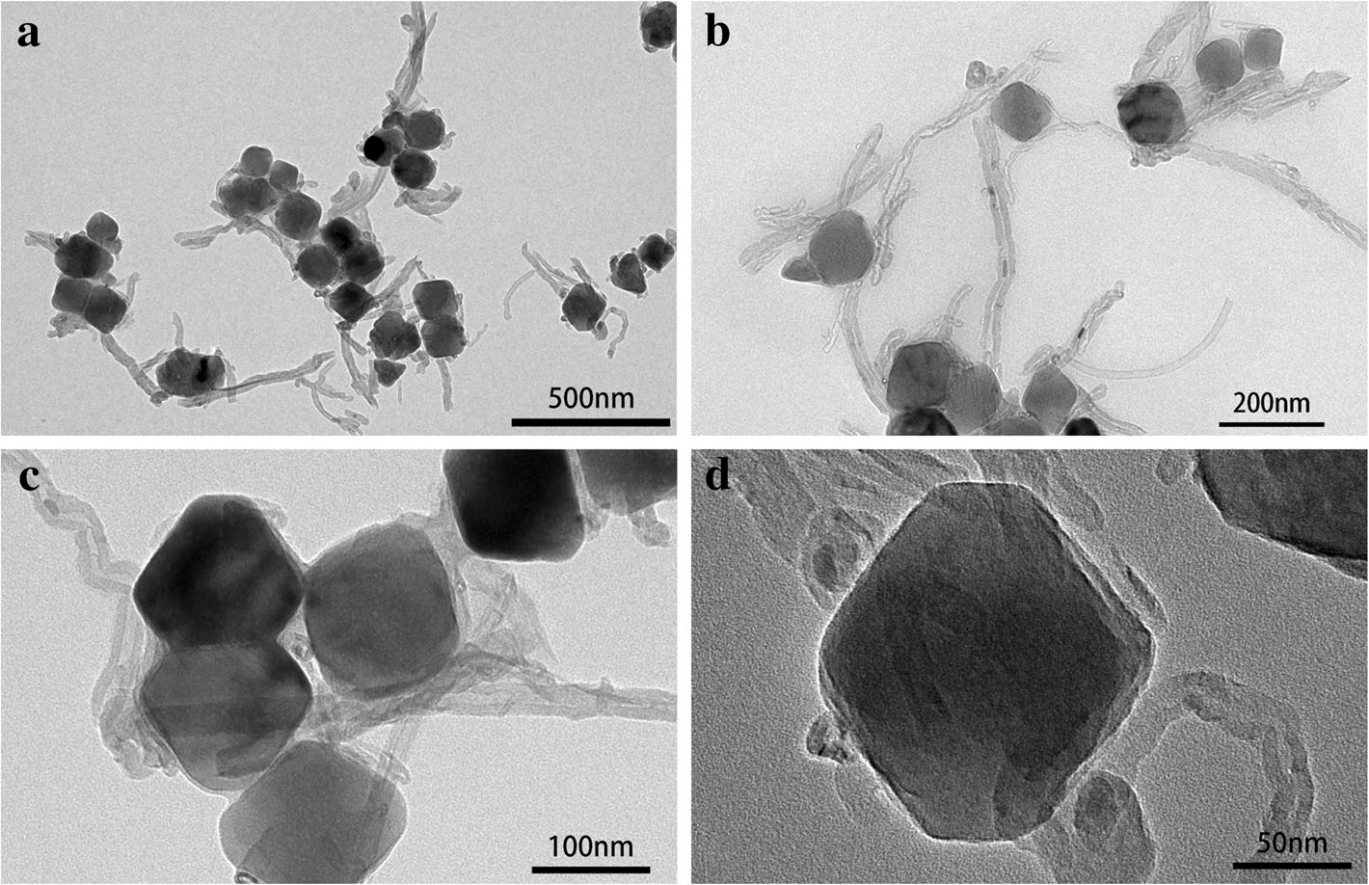
**a**–**d** TEM images of K(Mn_0.95_Ni_0.05_)F_3_/MWCNT composites corresponding to different magnifications

In order to improve the electronic conductivity of the material, K(Mn_0.95_Ni_0.05_)F_3_ was deposited on the MWCNTs to obtain K(Mn_0.95_Ni_0.05_)F_3_/MWCNT nanocomposites so as to obtain the excellent electrochemical performance. The formation of composite structure between K(Mn_0.95_Ni_0.05_)F_3_ and MWCNTs was confirmed by morphology and structure analysis. In Fig. 4, it was shown that the well-dispersed K(Mn_0.95_Ni_0.05_)F_3_ nanoparticles formed by bonding with MWCNTs. The size of the nanoparticles K(Mn_0.95_Ni_0.05_)F_3_ was still around the size range of 100, making it easy to form a good combination with nano-scale MWCNTs to further improve the conductivity of the material.

The chemical composition of K(Mn_0.95_Ni_0.05_)F_3_/MWCNTs was analyzed by XRD and XPS, and the results were shown in Additional file 1: Figure S1. The diffraction peak around 26° can be clearly seen in the XRD pattern of the sample of K(Mn_0.95_Ni_0.05_)F_3_/MWCNTs (Additional file 1: Figure S1a), verifying the presence of MWCNTs in the sample (JCPDS file No. 25-0284) [40]. XPS spectra were used to characterize the composition and the state of chemical bonding of the nanocomposite structure. The broad-spectrum scan confirmed the presence of K, F, Mn, Ni, and C elements in Additional file 1: Figure S1b. Aromatic carbon in MWCNTs was the most important source of C1s peak in the survey XPS spectrum [41], as shown clearly in Additional file 1: Figure S1c. In addition to the carbon in the MWCNT matrix, functional groups containing carbon and oxygen atoms (C=O and C–O) can also be obtained in Additional file 1: Figure S1c, demonstrating the presence of surface functional groups. Primarily, the high-resolution XPS spectrum of C1s depicted in Additional file 1: Figure S1c presented the chemical bonding C-F3 and C-F4 at 293.3 and 295.9 eV [42]. The formation of these bonds confirmed that fluorine of K(Mn_0.95_Ni_0.05_)F_3_ was attached to the carbon of the MWCNTs, thereby facilitating the achievement of good electron transfer between the active material and the conductive agent. The analysis herein proved that an effective bond was produced between the carbon nanotubes and the K(Mn_0.95_Ni_0.05_)F_3_ by chemical bonds.

### Electrochemical Performance as the Cathode of KIBs

**Fig. 5:**
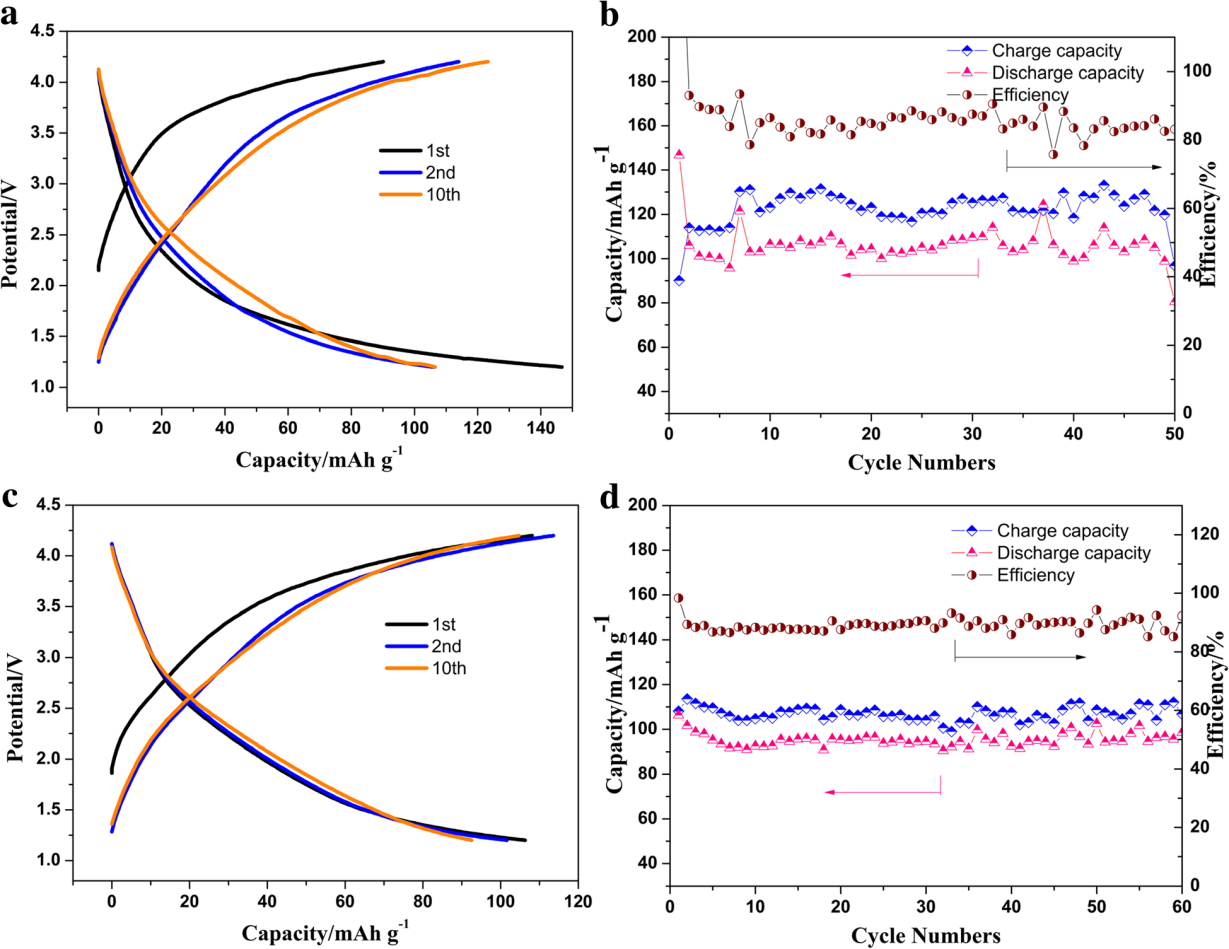
Charge–discharge profiles for the different cycles of K(Mn_0.95_Ni_0.05_)F_3_ (**a**) and K(Mn_0.95_Ni_0.05_)F_3_/MWCNTs (**c**), and the corresponding capacity-cycle profile of K(Mn_0.95_Ni_0.05_)F_3_ (**b**) and K(Mn_0.95_Ni_0.05_)F_3_/MWCNTs (**d**) at current density of 35 mA g^−1^ over the voltage range 4.2–1.2 V vs. K/K^+^

The electrochemical performances of the as-prepared material K(Mn_0.95_Ni_0.05_)F_3_ and K(Mn_0.95_Ni_0.05_)F_3_/MWCNTs were first evaluated to demonstrate the impact of MWCNTs addition. The galvanostatic charge/discharge cycling of K(Mn_0.95_Ni_0.05_)F_3_ and K(Mn_0.95_Ni_0.05_)F_3_/MWCNTs at a current density of 35 mA g^−1^ over the voltage range 4.2–1.2 V vs. K/K^+^ was shown in Fig. 5. From an overall view, the two materials exhibited high charge and discharge capacity due to better morphology control. Compared with K(Mn_0.95_Ni_0.05_)F_3_, the K(Mn_0.95_Ni_0.05_)F_3_/MWCNT electrode had a higher cycle stability and coulombic efficiency. During the first several cycles, the capacity of the K(Mn_0.95_Ni_0.05_)F_3_ electrode decreased monotonically, which might be ascribed to the SEI film stabilization and irreversible trapping of some potassium in the lattice [43]. Obviously, the charge–discharge capacity of K(Mn_0.95_Ni_0.05_)F_3_ showed obvious instability during the charge and discharge cycle, while the charge–discharge capacity of K(Mn_0.95_Ni_0.05_)F_3_/MWCNT material showed higher stability during 60 cycles. The charge and discharge capacities of K(Mn_0.95_Ni_0.05_)F_3_/MWCNTs after the 60th cycle can still reach 106.8 and 98.5 mAh g^−1^, respectively. A high capacity retention rate of 92.6% can still be maintained after 60 cycles. Since the basic materials and test conditions of the electrodes in these experiments were the same, we conclude that the improvement of the charge–discharge capacity of the battery results from the addition of MWCNTs.

The rate performance at different current densities of 35 mA g^−^1 to 280 mA g^−1^ was used to further evaluate the rate performance of the K(Mn_0.95_Ni_0.05_)F_3_/MWCNT cathode in the voltage range of 4.2–1.2 V. As shown in Additional file 1: Figure S2 a, the battery exhibited excellent cycle performance when the current density experienced different current densities. Additional file 1: Figure S2b presented the CV curves of K(Mn_0.95_Ni_0.05_)F_3_/MWCNT cathode at 0.2 mV s^−1^. The obtained CV curve was basically consistent with the charge and discharge process and also had the same characteristics as the CV curve of the sodium-ion battery of such materials. The CV curves were almost overlapped, implying the superior reversibility during deintercalation/intercalation process of K-ions.

### Electrochemical Impedance Spectroscopy of Synthetic Materials

**Fig. 6:**
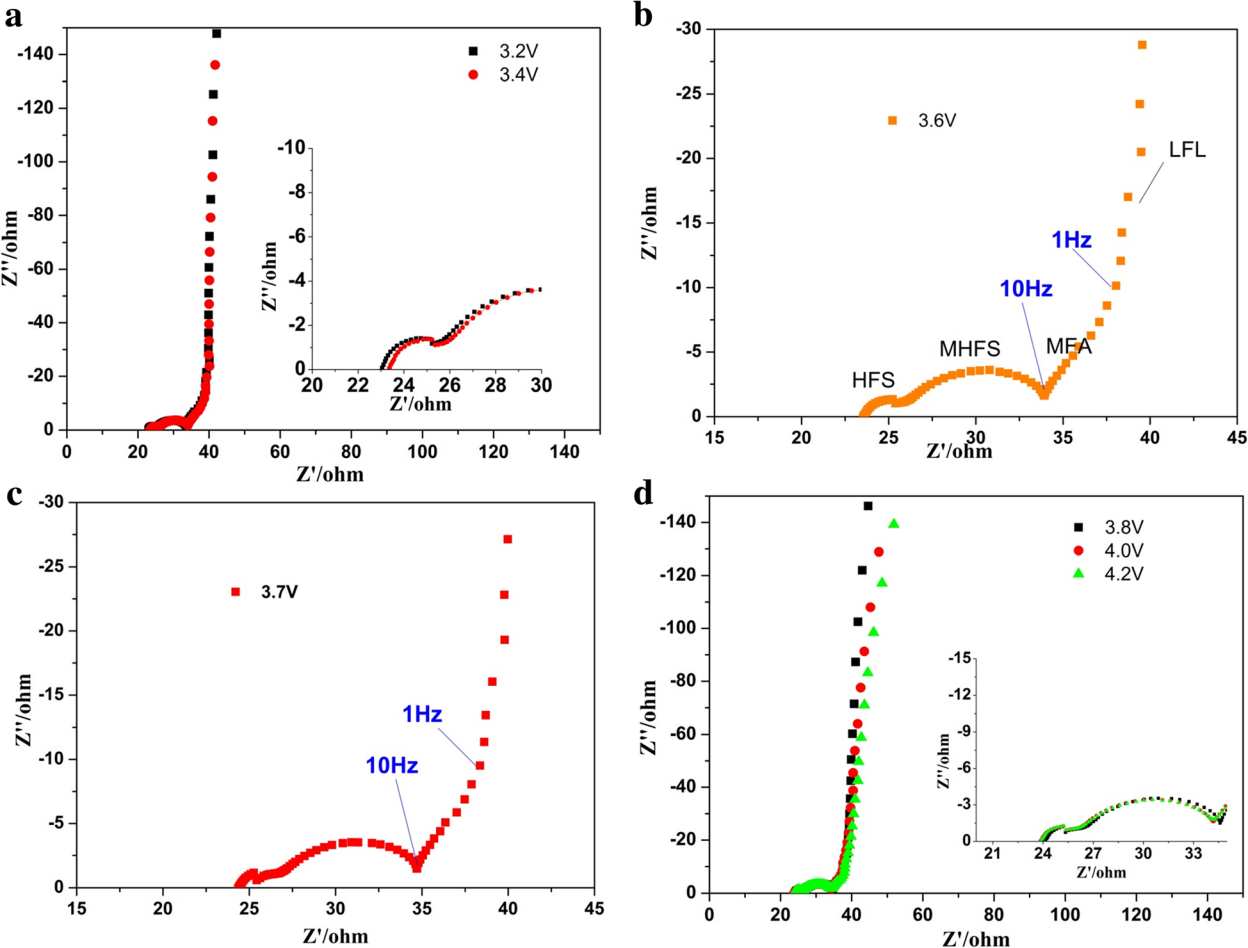
**a**–**d** Nyquist plots of the K(Mn_0.95_Ni_0.05_)F_3_/MWCNT electrode at various potentials during the first charge process

To investigate the interfacial reaction process of K(Mn_0.95_Ni_0.05_)F_3_/MWCNT composites at the electrode/electrolyte interface, EIS measurements of K(Mn_0.95_Ni_0.05_)F_3_/MWCNT composite electrodes were performed during the first charge and discharge process (Fig. 6 and Additional file 1: Figure S3). Under the open circuit potential, the Nyquist plots of K(Mn_0.95_Ni_0.05_)F_3_/MWCNTs during the first charge seem to consist of three components, namely, the high-frequency semicircle (HFS), the mid-frequency semicircle (MFS), and the mid-low frequency line or arc (MLFL/A). During the continuous increase of voltage to the end of charging and subsequent discharge, HFS and MFS were always present and did not change much. HFS was generally attributed to a semicircle associated with the formation of the SEI film. Combined with the charge and discharge process, it was known that the formation of the SEI film hardly occurred during the first cycle of charging, but occurred during the standing process before the charging process. The impedance spectrum after standing could prove the conclusion that the SEI film was formed at this stage (Additional file 1: Figure S4). This indicated that it was reasonable to have a semicircle associated with the SEI film at the open circuit voltage of the first cycle, and there would be no significant change during charging. This phenomenon further proved that HFS can be attributed to the migration of potassium-ion through SEI film [44]. The stable presence of the SEI film was one of the main reasons for the charge and discharge cycle stability of the composite electrode. According to the previous literature on fluoride EIS research [19], the MFS should be related to the Schottky contact between fluoride and conductive agents, which may be the important feature of such composite materials with the big band gap. Therefore, we can basically determine that MFS was related to electron conductivity. Combined with the frequency data given in Fig. 6b, c, it could be demonstrated that the cathode semicircle at several Hz (MLF) should be attributed to charge transfer [45]. The lower conductivity of the fluoride electrode resulted in a higher charge transfer resistance, so the semicircle of the mid-low frequency region only appeared as the line or arc. As the potential increased during charging, the low frequency region associated with the charge transfer process did not show the significant tendency to bend to form a circular arc, primarily due to the high charge transfer resistance [45–47]. Based on the above analysis, the three components that appear on the EIS spectrum were related to the SEI film, electron conductivity, and charge transfer resistance, respectively. The equivalent circuit for fitting the corresponding EIS diagram was shown in Additional file 1: Figure S5 and had typical characteristics of an equivalent circuit of a fluoride electrode material [48]. *R*_*s*_ stands for solution resistance, *R*_1_, *R*_2_, and *R*_3_, and constant phase angle elements (CPE; Q_1_, Q_2_, and Q_3_) represent the related resistors and capacitors of HFS, MFS, and LFS, respectively.

The Nyquist plots comparison of K(Mn_0.95_Ni_0.05_)F_3_ and K(Mn_0.95_Ni_0.05_)F_3_/MWCNT cathode at the first charge to 4.0 V was shown in Additional file 1: Figure S6. In the Nyquist diagram of KMnF_3_ to Ni^2+^/MWCNTs, the bending tendency of the oblique line in the MLF region representing the charge transfer process would be more pronounced. This also verified that the addition of MWCNTs improved the electrochemical activity of the positive electrode material to a certain extent, thereby improving the electrochemical performance. Since the region representing the charge transfer resistance still failed to bend into a semicircle, the charge transfer resistance might still be an important parameter affecting the electrochemical performance of the synthesized fluoride material.

## Conclusions

In summary, we reported the synthesis of concentration-gradient structure material K(Mn_0.95_Ni_0.05_)F_3_ and K(Mn_0.95_Ni_0.05_)F_3_/MWCNTs as cathode materials for KIBs. K(Mn_0.95_Ni_0.05_)F_3_ was synthesized by EDTA-assisted homogeneous precipitation method for the first time, and the formation process of concentration gradient of the material was predicted. This approach to prepare concentration-gradient structure fluoride cathode can be further extended to design other nano-structure systems for electrode material. On this basis, K(Mn_0.95_Ni_0.05_)F_3_ was deposited on the MWCNTs to improve the electron conductivity of the material so as to obtain the electrode material with more excellent electrochemical performance, such as charge–discharge capacity and cycle stability. As expected, the K(Mn_0.95_Ni_0.05_)F_3_/MWCNT composite electrode exhibited superb cycling stability. The charge and discharge capacities of K(Mn_0.95_Ni_0.05_)F_3_/MWCNTs after the 60th cycle can still reach 106.8 and 98.5 mAh g^−1^ over the voltage range 4.2–1.2 V vs. K/K^+^ at the current density of 35 mA g^−1^. The Nyquist diagram of K(Mn_0.95_Ni_0.05_)F_3_/MWCNT composite electrode revealed that charge transfer resistance might be an important parameter affecting the electrochemical performance of the synthetic fluoride material.

## Additional file

Additional file 1: **Figure S1.** K(Mn_0.95_Ni_0.05_)F_3_/MWCNT composites. (a) XRD pattern, (b) XPS survey spectrum, (c) high-resolution XPS spectrum of C1s. **Figure S2.** Rate performance (a) and CV curves (b) of K(Mn_0.95_Ni_0.05_)F_3_/MWCNTs as the cathode over the voltage range 4.4–1.2 V vs. K/K^+^. **Figure S3.** Nyquist plots of the K(Mn_0.95_Ni_0.05_)F_3_/MWCNT electrode at various potentials during the first discharge process. **Figure S4.** Nyquist plots of the K(Mn_0.95_Ni_0.05_)F_3_/MWCNT electrode at Open circuit potential. **Figure S5.** Equivalent circuit of K(Mn_0.95_Ni_0.05_)F_3_/MWCNT cathode during the first charge and discharge process. **Figure S6.** Nyquist plots of K(Mn_0.95_Ni_0.05_)F_3_ and K(Mn_0.95_Ni_0.05_)F_3_/MWCNT cathode at the first charge to 4.0 V. (ZIP 4678 kb)

## Data Availability

The data supporting the conclusions of this article are included within the article.

## Abbreviations

CPE: Constant phase angle elements
DEC: Diethyl carbonate
EC: Ethylene carbonate
EDS: Energy-dispersive spectrum
EIS: Electrochemical impedance spectroscopy
HFS: High-frequency semicircle
ICP-AES: Inductively coupled plasma atomic emission spectrometry
KIBs: Potassium-ion batteries
LIBs: Lithium-ion batteries
MFS: Mid-frequency semicircle
MLFL/A: Mid-low frequency line or arc
MWCNTs: Multi-walled carbon nanotubes
NIBs: Sodium-ion batteries
NMP: *N*-Methyl pyrrolidinone
PVDF: Polyvinylidene fluoride
TEM: Transmission electron microscopy
XRD: X-ray diffraction
