# Supplementary figures and images for: Scalable Synthesis Nano-Perovskite K(Mn0.95Ni0.05)F3 Cathode by Homogeneous Precipitation Method for Potassium-Ion Batteries
Source: Nanoscale Res Lett. 2019 Jul 16;14:238. doi: 10.1186/s11671-019-3056-1 (PMC6635574; doi:10.1186/s11671-019-3056-1)

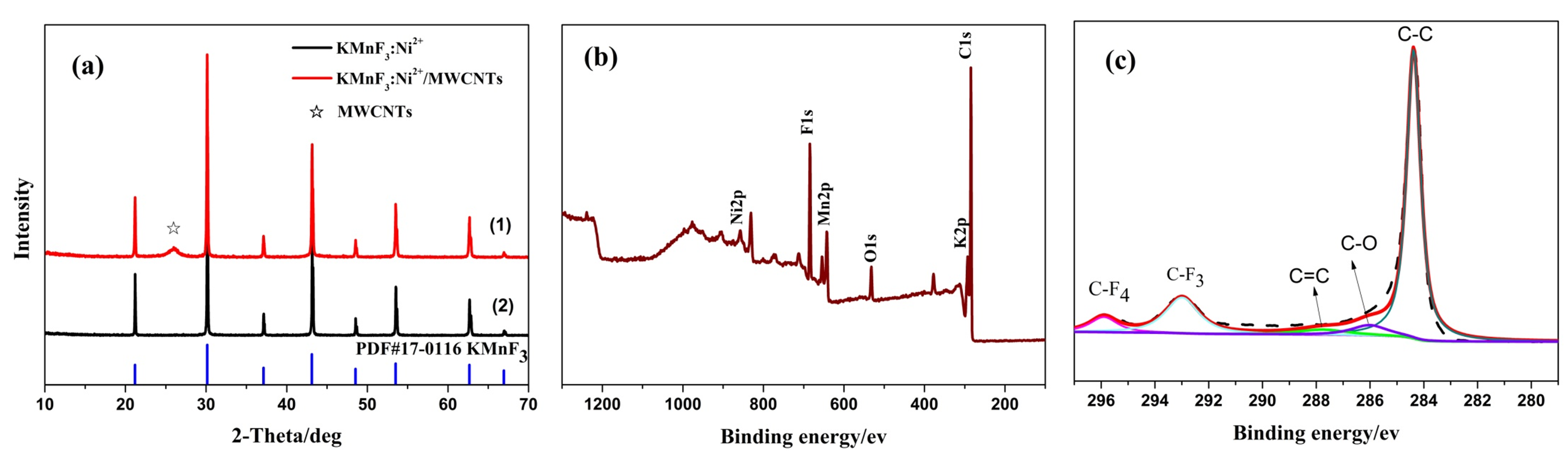

Supplement: Supplementary file 1 — Figure S1. K(Mn0.95Ni0.05)F3/MWCNT composites. (a) XRD pattern, (b) XPS survey spectrum, (c) high-resolution XPS spectrum of C1s. Figure S2. Rate performance (a) and CV curves (b) of K(Mn0.95Ni0.05)F3/MWCNTs as the cathode over the voltage range 4.4–1.2 V vs. K/K+. Figure S3. Nyquist plots of the K(Mn0.95Ni0.05)F3/MWCNT electrode at various potentials during the first discharge process. Figure S4. Nyquist plots of the K(Mn0.95Ni0.05)F3/MWCNT electrode at Open circuit potential. Figure S5. Equivalent circuit of K(Mn0.95Ni0.05)F3/MWCNT cathode during the first charge and discharge process. Figure S6. Nyquist plots of K(Mn0.95Ni0.05)F3 and K(Mn0.95Ni0.05)F3/MWCNT cathode at the first charge to 4.0 V. (ZIP 4678 kb) [file 11671_2019_3056_MOESM1_ESM.zip › Fig S1.tif]

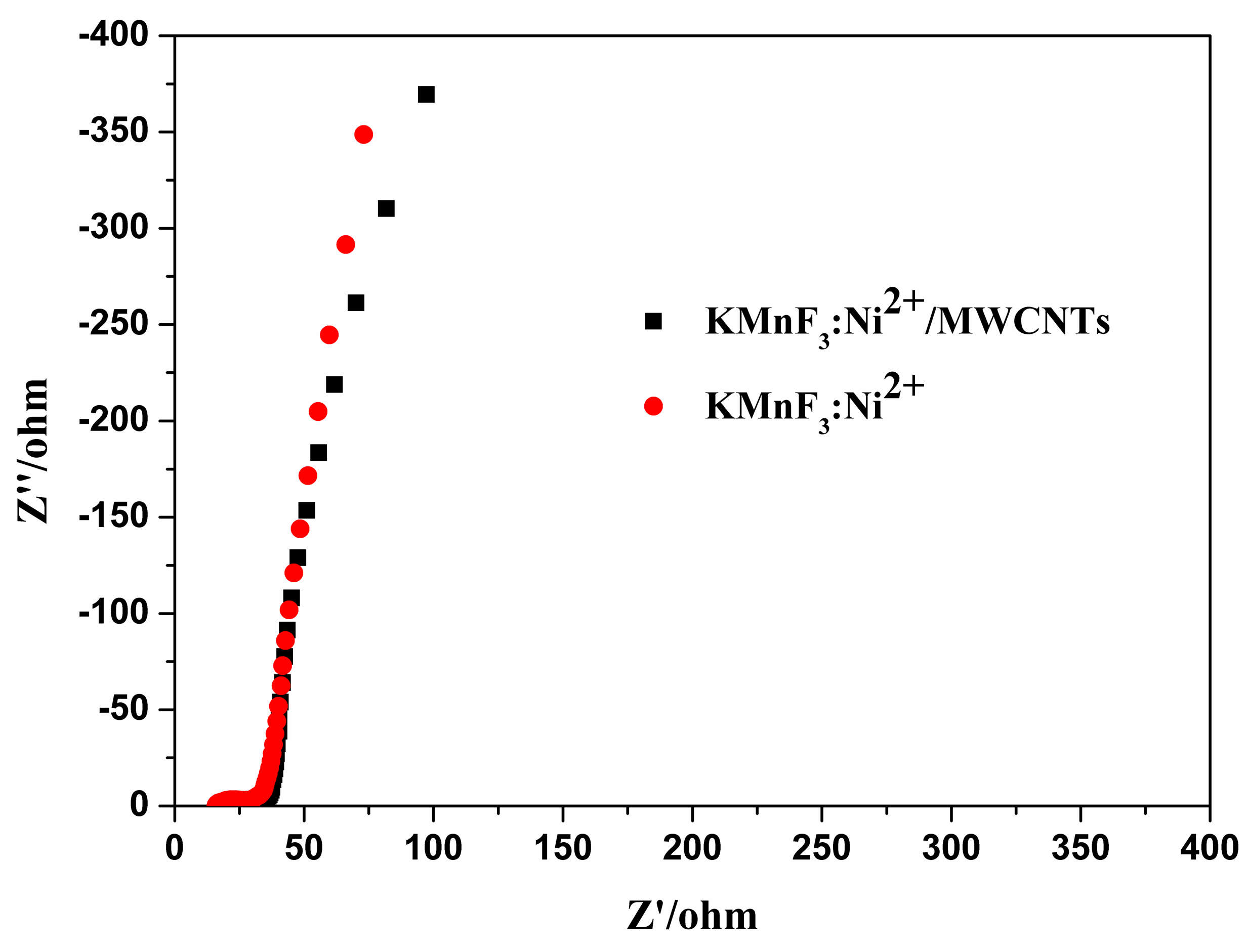

Supplement: Supplementary file 1 — Figure S1. K(Mn0.95Ni0.05)F3/MWCNT composites. (a) XRD pattern, (b) XPS survey spectrum, (c) high-resolution XPS spectrum of C1s. Figure S2. Rate performance (a) and CV curves (b) of K(Mn0.95Ni0.05)F3/MWCNTs as the cathode over the voltage range 4.4–1.2 V vs. K/K+. Figure S3. Nyquist plots of the K(Mn0.95Ni0.05)F3/MWCNT electrode at various potentials during the first discharge process. Figure S4. Nyquist plots of the K(Mn0.95Ni0.05)F3/MWCNT electrode at Open circuit potential. Figure S5. Equivalent circuit of K(Mn0.95Ni0.05)F3/MWCNT cathode during the first charge and discharge process. Figure S6. Nyquist plots of K(Mn0.95Ni0.05)F3 and K(Mn0.95Ni0.05)F3/MWCNT cathode at the first charge to 4.0 V. (ZIP 4678 kb) [file 11671_2019_3056_MOESM1_ESM.zip › Fig S6.tif]

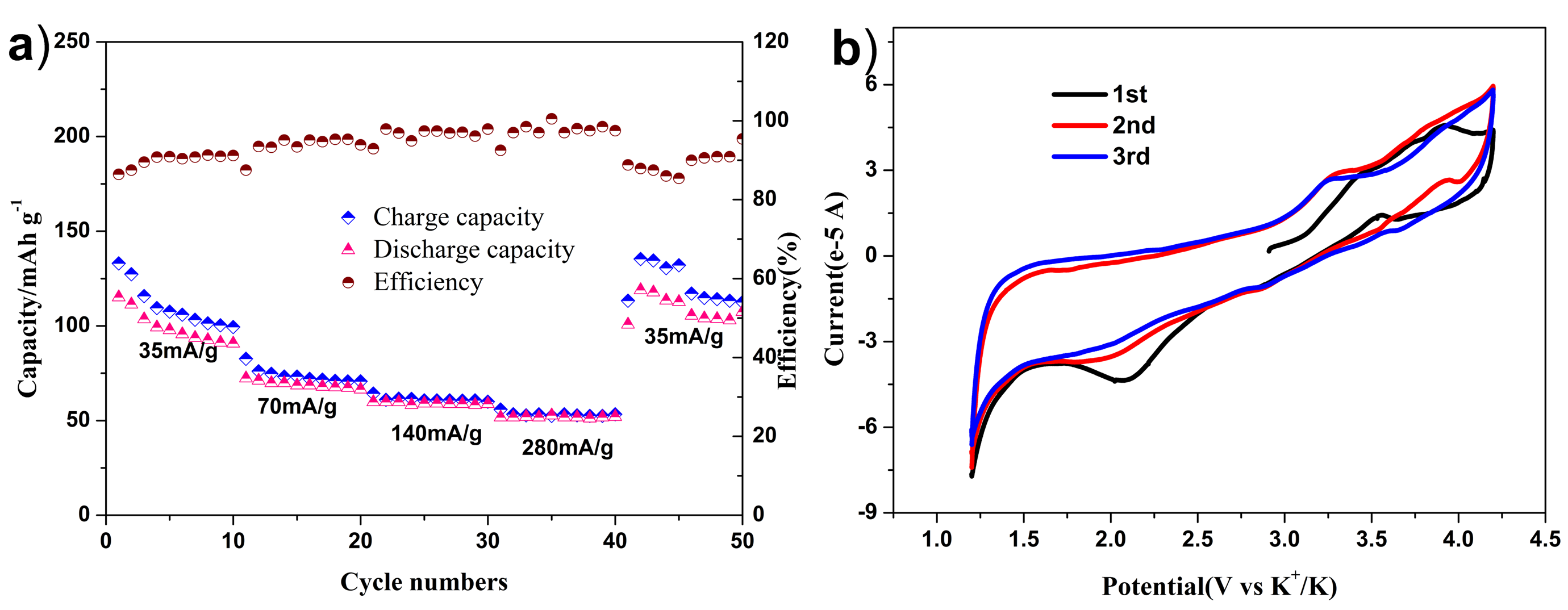

Supplement: Supplementary file 1 — Figure S1. K(Mn0.95Ni0.05)F3/MWCNT composites. (a) XRD pattern, (b) XPS survey spectrum, (c) high-resolution XPS spectrum of C1s. Figure S2. Rate performance (a) and CV curves (b) of K(Mn0.95Ni0.05)F3/MWCNTs as the cathode over the voltage range 4.4–1.2 V vs. K/K+. Figure S3. Nyquist plots of the K(Mn0.95Ni0.05)F3/MWCNT electrode at various potentials during the first discharge process. Figure S4. Nyquist plots of the K(Mn0.95Ni0.05)F3/MWCNT electrode at Open circuit potential. Figure S5. Equivalent circuit of K(Mn0.95Ni0.05)F3/MWCNT cathode during the first charge and discharge process. Figure S6. Nyquist plots of K(Mn0.95Ni0.05)F3 and K(Mn0.95Ni0.05)F3/MWCNT cathode at the first charge to 4.0 V. (ZIP 4678 kb) [file 11671_2019_3056_MOESM1_ESM.zip › Fig.S2.tif]

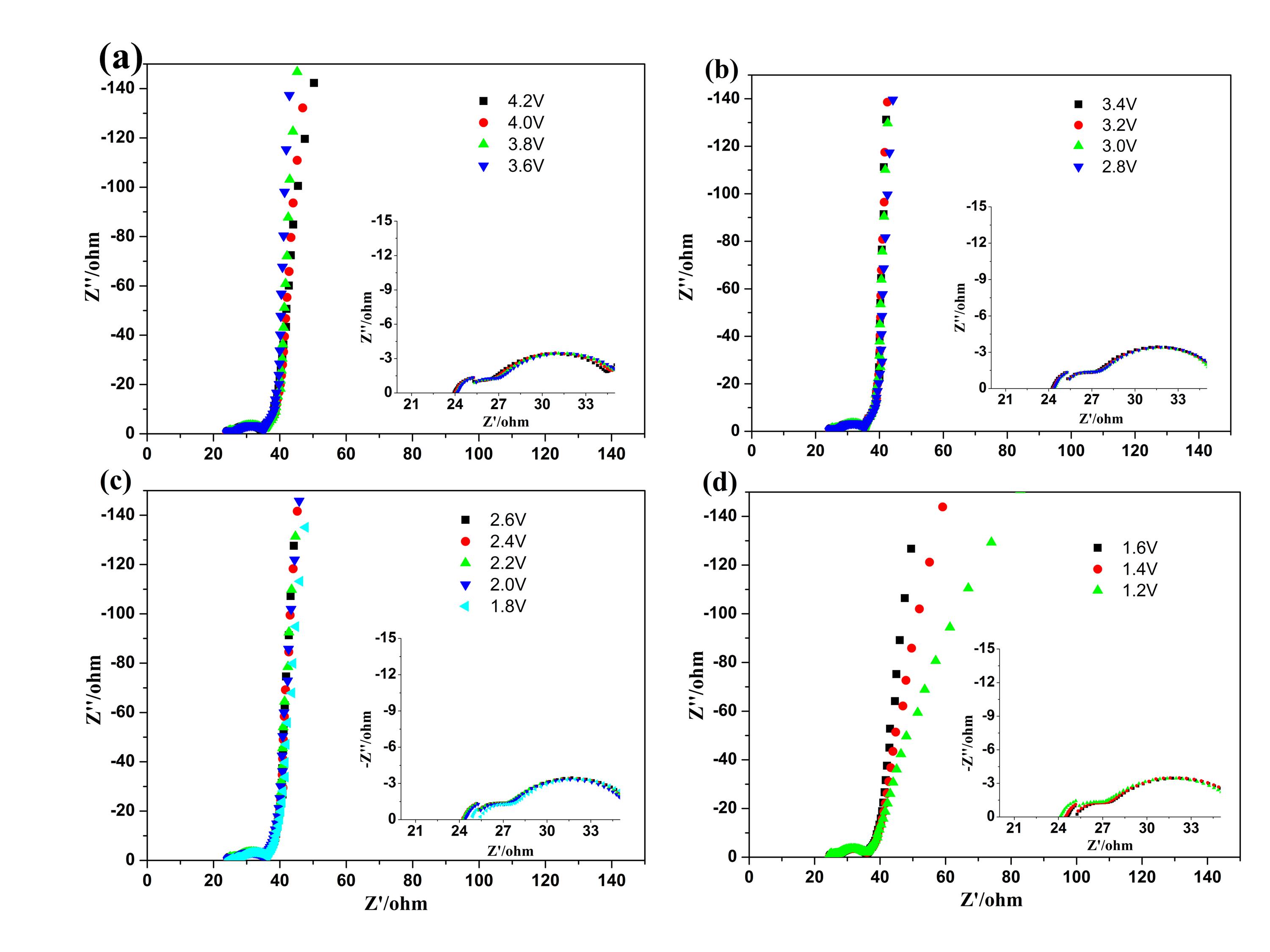

Supplement: Supplementary file 1 — Figure S1. K(Mn0.95Ni0.05)F3/MWCNT composites. (a) XRD pattern, (b) XPS survey spectrum, (c) high-resolution XPS spectrum of C1s. Figure S2. Rate performance (a) and CV curves (b) of K(Mn0.95Ni0.05)F3/MWCNTs as the cathode over the voltage range 4.4–1.2 V vs. K/K+. Figure S3. Nyquist plots of the K(Mn0.95Ni0.05)F3/MWCNT electrode at various potentials during the first discharge process. Figure S4. Nyquist plots of the K(Mn0.95Ni0.05)F3/MWCNT electrode at Open circuit potential. Figure S5. Equivalent circuit of K(Mn0.95Ni0.05)F3/MWCNT cathode during the first charge and discharge process. Figure S6. Nyquist plots of K(Mn0.95Ni0.05)F3 and K(Mn0.95Ni0.05)F3/MWCNT cathode at the first charge to 4.0 V. (ZIP 4678 kb) [file 11671_2019_3056_MOESM1_ESM.zip › Fig.S3.tif]

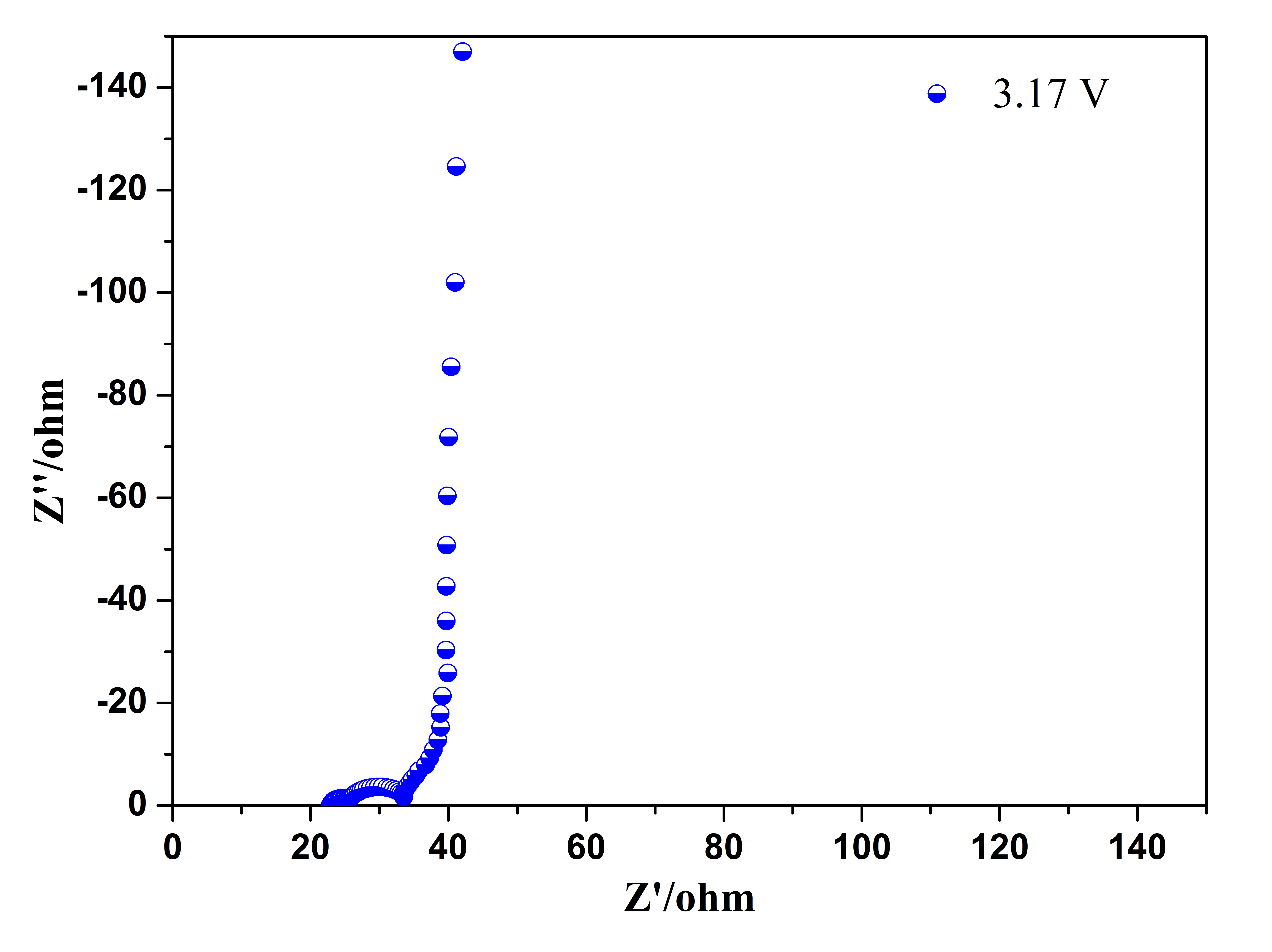

Supplement: Supplementary file 1 — Figure S1. K(Mn0.95Ni0.05)F3/MWCNT composites. (a) XRD pattern, (b) XPS survey spectrum, (c) high-resolution XPS spectrum of C1s. Figure S2. Rate performance (a) and CV curves (b) of K(Mn0.95Ni0.05)F3/MWCNTs as the cathode over the voltage range 4.4–1.2 V vs. K/K+. Figure S3. Nyquist plots of the K(Mn0.95Ni0.05)F3/MWCNT electrode at various potentials during the first discharge process. Figure S4. Nyquist plots of the K(Mn0.95Ni0.05)F3/MWCNT electrode at Open circuit potential. Figure S5. Equivalent circuit of K(Mn0.95Ni0.05)F3/MWCNT cathode during the first charge and discharge process. Figure S6. Nyquist plots of K(Mn0.95Ni0.05)F3 and K(Mn0.95Ni0.05)F3/MWCNT cathode at the first charge to 4.0 V. (ZIP 4678 kb) [file 11671_2019_3056_MOESM1_ESM.zip › Fig.S4.tif]

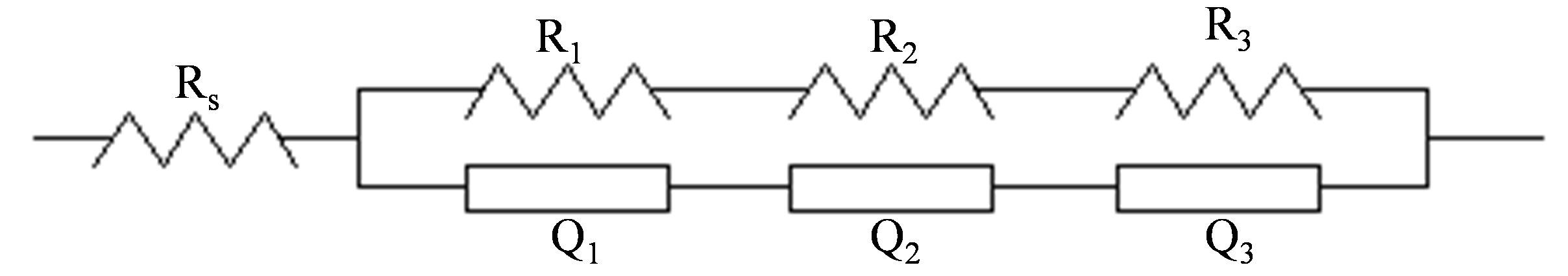

Supplement: Supplementary file 1 — Figure S1. K(Mn0.95Ni0.05)F3/MWCNT composites. (a) XRD pattern, (b) XPS survey spectrum, (c) high-resolution XPS spectrum of C1s. Figure S2. Rate performance (a) and CV curves (b) of K(Mn0.95Ni0.05)F3/MWCNTs as the cathode over the voltage range 4.4–1.2 V vs. K/K+. Figure S3. Nyquist plots of the K(Mn0.95Ni0.05)F3/MWCNT electrode at various potentials during the first discharge process. Figure S4. Nyquist plots of the K(Mn0.95Ni0.05)F3/MWCNT electrode at Open circuit potential. Figure S5. Equivalent circuit of K(Mn0.95Ni0.05)F3/MWCNT cathode during the first charge and discharge process. Figure S6. Nyquist plots of K(Mn0.95Ni0.05)F3 and K(Mn0.95Ni0.05)F3/MWCNT cathode at the first charge to 4.0 V. (ZIP 4678 kb) [file 11671_2019_3056_MOESM1_ESM.zip › Fig.S5.tif]
